# Do-it-yourself methodology for calorimeter construction based in Arduino data acquisition device for introductory chemical laboratories

**DOI:** 10.1016/j.heliyon.2020.e03591

**Published:** 2020-03-18

**Authors:** William Vallejo, Carlos Diaz-Uribe, Catalina Fajardo

**Affiliations:** Grupo de Fotoquímica y Fotobiología, Universidad del Atlántico, Carrera 30 Número 8- 49 Puerto Colombia, Barranquilla, Colombia

**Keywords:** Physical chemistry, Education, Experimental class, Computer-based learning, Laboratory computing, Secondary education, First -year undergraduate

## Abstract

Many experimental thermochemical laboratories require monitoring temperatures during a reaction or physical procedure. Nowadays, there are many alternatives to fulfill this requirement; however, they are expensive for basic scholars and first-year undergraduates. In this paper, we describe an inexpensive and useful data acquisition device developed with the open-source Arduino software. In this work, we presented a methodology for easy calorimeter construction based in Arduino data acquisition device for introductory chemical laboratories, we used an LM35 transistor as a temperature sensor connected to an Arduino UNO microcontroller for temperature sensing and an aquarium air pump for agitation of reaction system. Besides, the hardware required for implementation is explained in detail. The device was built using the (DIY) do-it -yourself method, and the complete system had a total cost under $40. We showed details of all components for data acquisition construction. Finally, we tested the device in order to determine the exothermic dissolution heat (ΔH) for NaOH in water.

## 1 Introduction

Experimental courses permit students to consolidate and apply theoretical knowledge obtained in other courses (Chen et al., 2013; Tobajas et al., 2018). Experimental courses are present in both secondary courses and all academic years of degree programs and they require novel strategies to improve skills of students. In situ data acquisition in laboratories give to students information to study and understand physical chemical phenomenon and the data acquisition during reaction require to develop efficient tools for studying simples systems (Molina et al., 2018; Manafov, 2015). Nowadays, data acquisition devices are important teaching tools in secondary and besides for chemistry and chemical engineering laboratories; however, these systems are expensive and out of the scope of traditional basic scholars and general chemistry programs. The market offer data acquisition systems at a price between $100 and $500 (National Instruments, 2020b), and propriety software $1300 - $6000 for professional packages (National Instruments, 2020a). For universities, the market offers package annual fees of $100 per license allowing the use of the most current professional development system software (Caltech, 2015).

The Open-source microelectronics have two main characteristics that have made these devices highly useful in different chemistry laboratories: (a) hardware costs are lower than traditional interfaces, and (b) the open-source software is free of charge (Grinias et al., 2016; Urban, 2014). Arduino has recently become a quite popular microcontroller (e.g. the most popular one is Arduino Uno). Arduino is an open electronic platform for the creation of prototypes based on both free software and low-cost hardware, the program is written in the C++ code.

In recent years, the use of Arduino-based instrumentation has increased in instrumental laboratories: (i) polymerase chain reaction (PCR) thermocyclers (Mabbott, 2014), (ii) automated burets (Famularo et al., 2016), (iii) photometers (McClain, 2014), (iv) thermometers and PH-meters (Kubínová and Šlégr, 2015). All these recent reports verify the potential of Arduino software and hardware. Currently The reform of chemistry curriculum is both promising and challenging as past teaching methods are examined in light of more current educational goals (Tenaw, 2015). Information and communication technology and DIY methodology open up a new educational world of creativity for students and teachers, these strategies for teaching chemistry plays an important role in planning lessons and in their management (Grimaldi and Rapuano, 2009; Tenaw, 2015).

Last decades, several authors reported calorimeters made from alternative materials. Stankus et al., constructed calorimeters with polypropylene Tri-Pour beakers for determining the enthalpy of solution of sodium hydroxide (Stankus and Caraway, 2011), Ruekberg reported calorimeters made from Thermos brand snack jars (Ruekberg, 1994), Kavanagh et al., used calorimeters made from glass beakers (Kavanagh et al., 2008); Bopegedera et al. reported the construction of a Coffee cup calorimetry, for using a Vernier temperature probe and LoggerPro data collection software Vernier Software & Technology (Vernier, 2019), although these are viable alternatives, the initial cost of their construction could be prohibitive for institutions with limited budgets (Bopegedera and Perera, 2017).

In the present report, we describe an easy calorimeter construction using DIY methodology and Arduino-based circuit for electronic temperature sensing and data acquisition. Besides, we checked the device to determine the exothermic dissolution enthalpy (ΔH) of NaOH(s) in water. We provided all the instructions necessary to the software and hardware construction. This device is an inexpensive and stable alternative for USB-driven data acquisition for undergraduates in introductory thermochemical lab classes.

## 2 Experimental

We utilized Arduino Uno (ATmega328, chip FTDI to USB/series signal conversion), a breadboard and LM35 transistor for control and data acquisition. The LM35 transistor, the Arduino Uno microcontroller and the breadboard are wired via jumper cables. The LM35 is adapted and protected via heat shrinkable sleeves and jumper cables and besides for agitation of reaction system we used an aquarium air pump. In next section, we present a complete description of the circuit diagram and the step-by-step wiring process to construct device. The device's total costs did not exceed $40, which is one-half to one-tenth the cost of sensors with interfaces available on the market. Although the devise's USB data acquisition rate is slower than that of some commercial USB data acquisition setups available on the market, it is suitable for most introductory thermochemical laboratories. Furthermore, the main advantage of the device is the Arduino software; it is open-source software, and it can be installed without any license, which notably reduces costs.

### 2.1 Temperature sensing wiring

**Figure 1:**
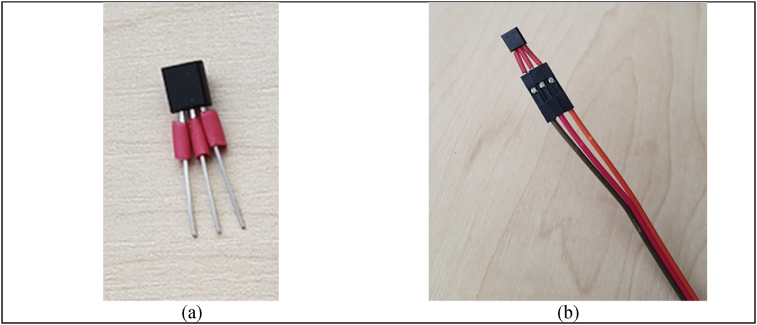
(a) Photography of the step 1 for protecting pin of the lm35. (b) photography of the step 2 for temperature sensor wiring.

**Figure 2:**
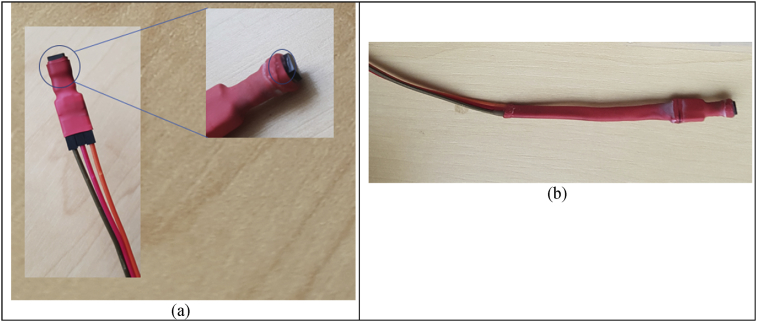
(a) photography of the step 3 for protecting the lm35 transistor. (b) photography of the step 4 for protecting the temperature sensor wiring.

The LM35 is an integrated-circuit with an output voltage linearly proportional to the centigrade temperature. You find complete datasheet in reference (Texas Instruments, 1999). Next we detail the step-by-step procedure to arrange LM35 (see Figures 1 and 2):

1. Put a heat shrinkable sleeve around each pin of LM35 (Figure 1a)
2. Connect each pin del LM35 to a jumper wire (Figure 1b)
3. Put a heat shrinkable sleeve around LM35 and Jumper wire connection. After that heat up gently shrinkable sleeve, to protect LM35 you must seal the top part applying a small amount of epoxy resin (e.g. UHU®, Pegadit®), (Figure 2a).
4. Put a heat shrinkable sleeve around LM35 and jumper. After that heat up gently shrinkable sleeve (Figure 2b).
5. Connect other jumper wire to increase the length of the device. In this part, you are ready to connect the device to Arduino UNO board.

### 2.2 Connection to Arduino board

**Figure 3:**
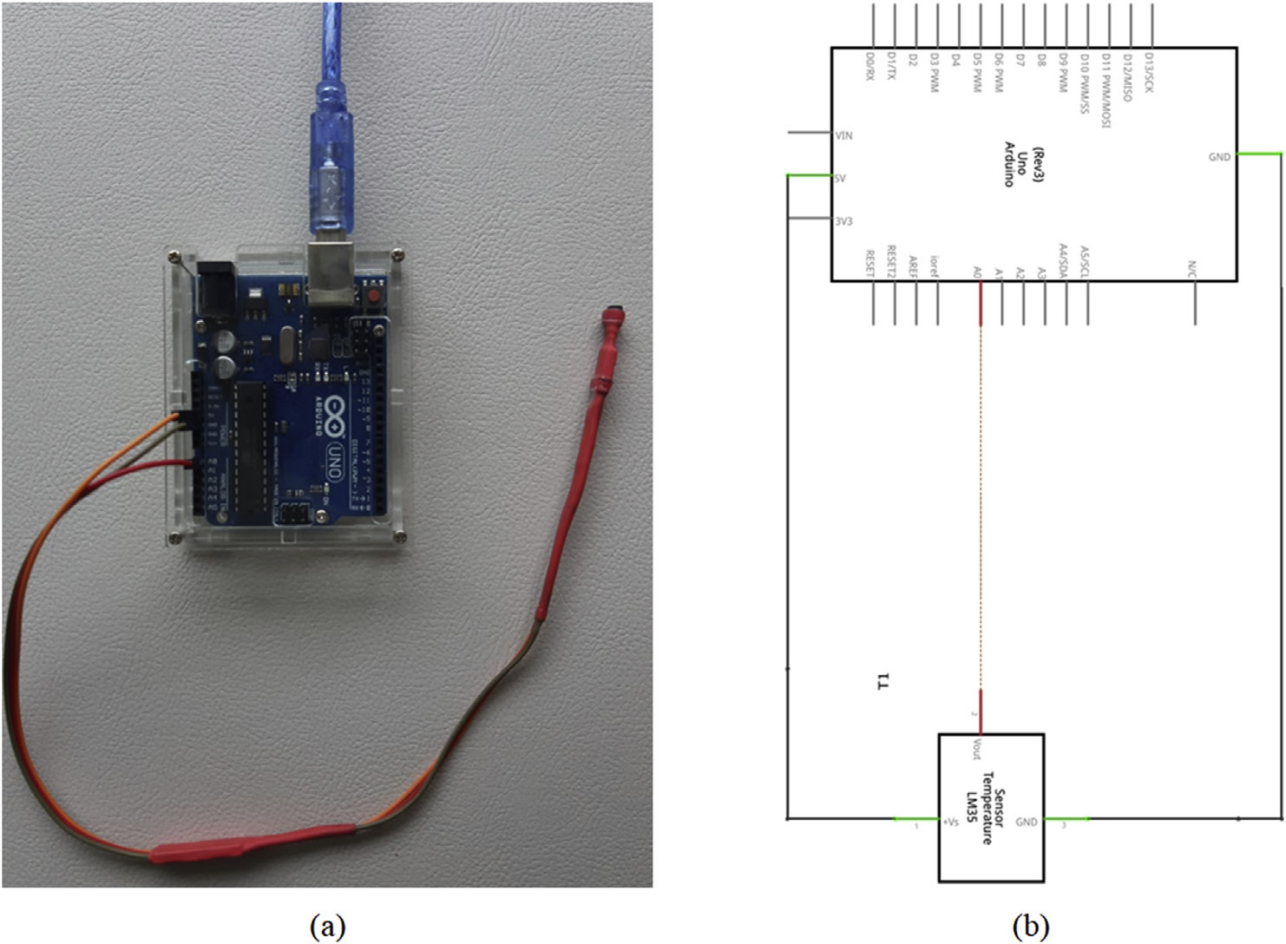
(a) LM35 connection to Arduino board photography. (b) scheme for circuit for photography of the step 2 for Temperature sensing device (circuit was created by Fritzing open-source software, Fritzing, 2018).

We used Arduino UNO board. You can buy it for $10–20 on any online-store (e.g. Mercado libre, eBay); you can also use a clone of Arduino board that is cheaper than original board. Figure 3(a) shows photography of the complete circuit. We used Fritzing software to building the circuit and connections Arduino UNO board and LM35 (Figure 3b). Fritzing software is an open-source hardware initiative that makes electronics accessible as a creative material for anyone, you can download from Fritzing homepage (Fritzing, 2018).

The LM35 V_out_ pin is connected to the analogic pin (A0) of the Arduino board, the LM35 Ground pin is connected to the ground of Arduino board and LM35 voltage source is connected to 5 V channel of Arduino board. When the LM35 and Arduino board are connected (figure 3a,b), connect the device to PC via Arduino USB port.

### 2.3 Sketch code installation

**Figure 4:**
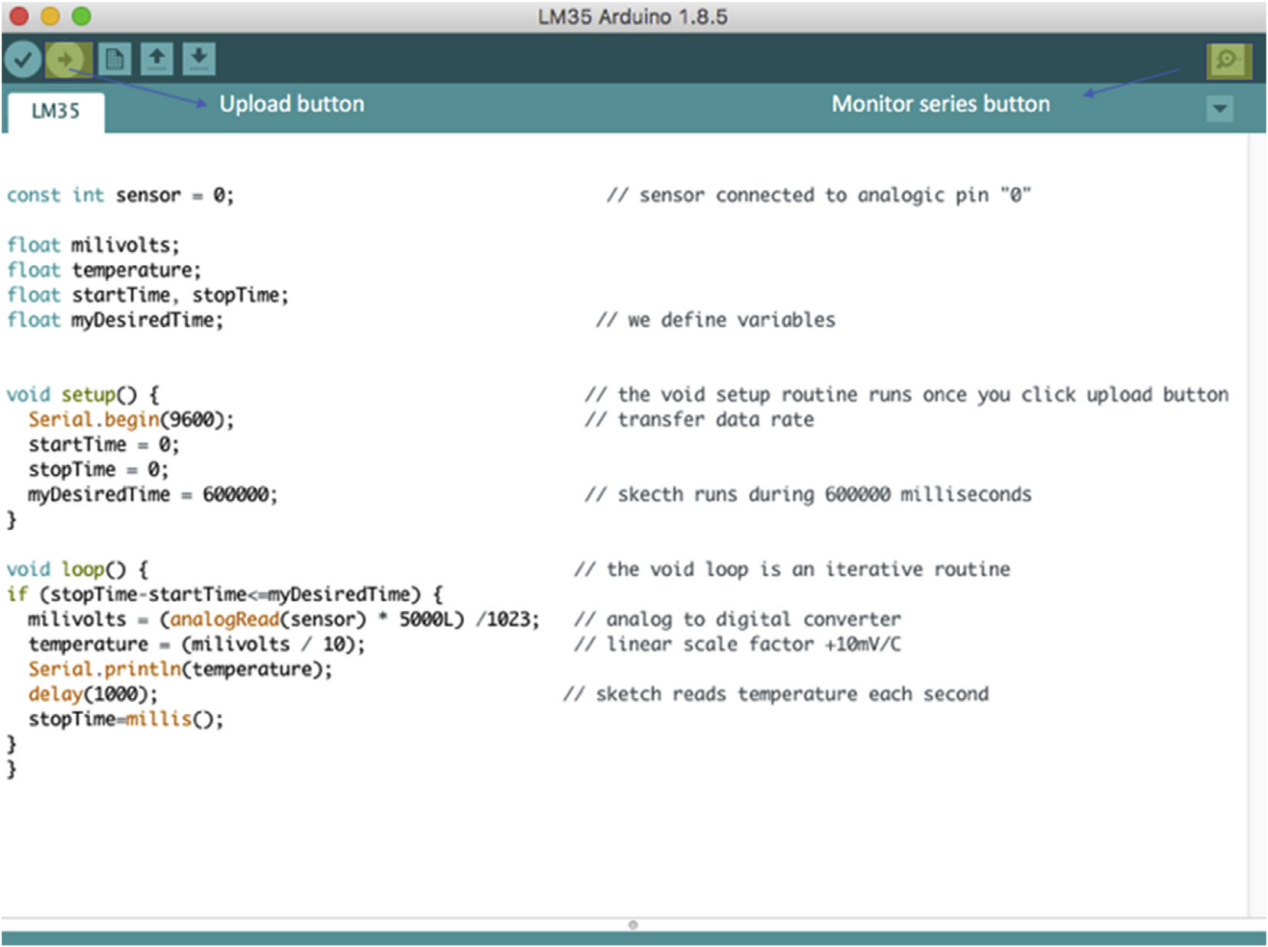
Sketch code to data temperature acquisition, inside sketch you find a reason for each part of code (see supplementary information for sketch code).

The open-source Arduino Software makes it easy to write code and upload it to the board. It runs on Windows, Mac OS X, and Linux. You can download from Arduino homepage (Arduino Home Page, 2018c). After downloading the software, you can run the installation software for Arduino application according to your software operating systems (MacOS, Windows or Linux). After installation, you must install library titled “LM35” (library is the name to files in Arduino software), you can download library from supporting information. After that, you must open file titled “LM35” and you will see the sketch shown in Figure 4 (sketch is the name to code in Arduino software). The LM35 sensor is wiring to pin zero (A0) of the Arduino microcontroller. The code runs only after user click on “upload” button (it is highlighted in Figure 4) and it will stop after 600000 ms (“myDesiredTime = 600000” by default) but you can change it according to your needs. you only need to rewrite the code according to the time you required. The “analogRead” function reads the value from the specified analog pin, for our case pin where sensor is connected. The input voltages in Arduino boards are divided into levels between 0 and 1023 (Arduino Home Page, 2018a). The “anologRead() function” requires a formula converter to read a correct temperature (see Figure 1). Furthermore, the transistor LM35 has a linear +10mV/^O^C scale factor (Combining the resolution of the A/D converter with the 10 mV/◦C gain factor of the LM35, the device has a rough resolution of ±0.5 °C per bit). Finally, sketch reads temperature every 1000 ms (“delay 1000” by default) but you can change it according to your need. The “millis()” function returns the number of milliseconds since the Arduino board began running the program; you can view data acquisition after clicking on the “Monitor series” button (Arduino Home Page, 2018b).

**Figure 5:**
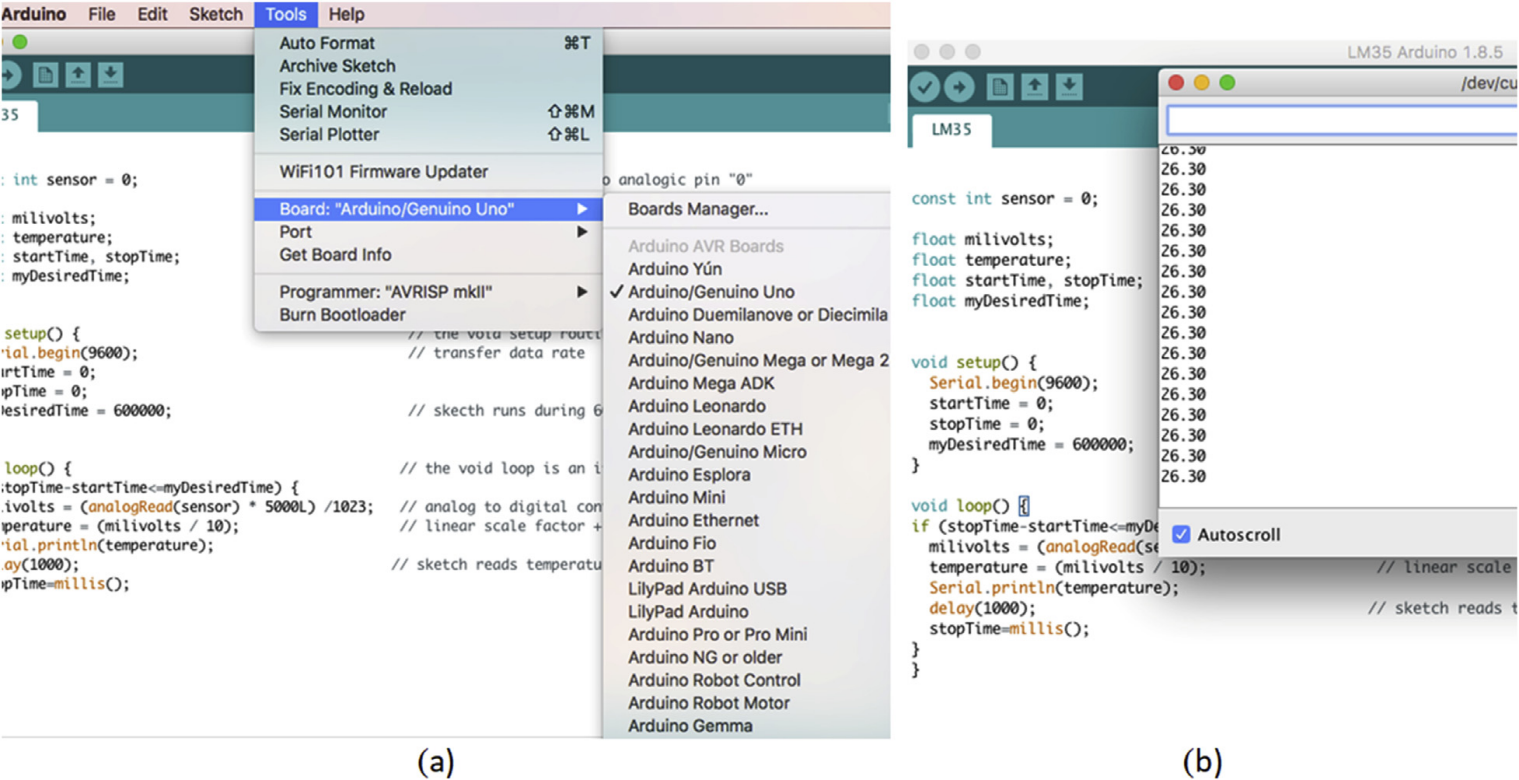
(a) route to verify PC and Arduino board connection, (b) view after you run code.

The sketch permits: (a) to change time delay data; (b) change the time of data acquisition; and (c) data measurements are sent to a PC via an USB port. Finally, the sketch can be rewritten to improve data acquisition (Monk, 2012). Before starting to run the code, you must verify the connection between PC and Arduino board. For that, you proceed to open file titled “LM35” and verify connection (Tool/Board/Arduino Uno). Figure 5(a) shows the route to verify the connection.

### 2.4 Calorimeter construction and testing device

In chemistry-introductory labs is common to use an open Styrofoam cup as a solution calorimeter, however, different authors have reported this device can present substantial heat loss due to evaporation and besides (Wong et al., 2001). Kavanagh et al. indicated that the solutions may not be at the same temperature when they are mixed with each other inside of Styrofoam cup (Kavanagh et al., 2008).

**Figure 6:**
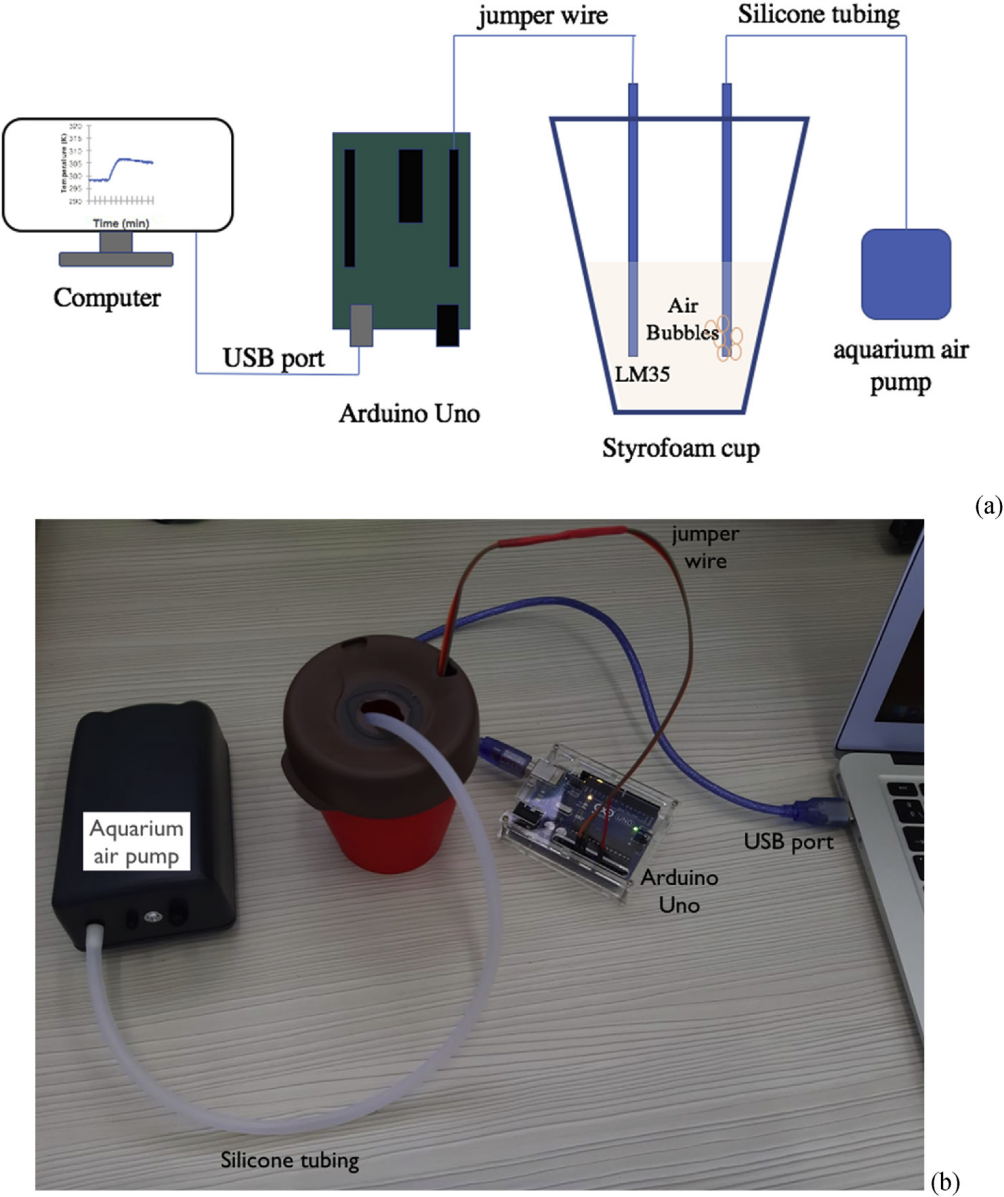
(a) General scheme of calorimeter fabricated if this work. (b) photography of calorimeter fabricated if this work.

Usually, the solution mixing is carried out by magnetic stirring, however, when this equipment is not available mixing is carried out by manual stirring. We tried to address these requirements for using an aquarium air pump for agitation of reaction system, the air pump is connecting by a silicone tubing to the double Styrofoam cup and the, the rate of bubbling is controlled easily by limiting the size of the silicone tubing tip immersing inside Styrofoam cup see Figure 6. The Figure 6 shows the general scheme of the calorimeter device, both the sensing temperature device (section 2.1) and the aquarium air pump are connected to the Styrofoam cup.

After you have verified connection between PC and Arduino board and turned on the aquarium air pump, you must to immerse sensor into solution, then click on upload button to upload the code to Arduino board (Figure 4). This process begins communication between PC and Arduino board. If code is uploaded suitably; then data acquisition will start and you would see data acquisition in real time after clicking on monitor series button (see Figure 5b). When data acquisition has finished, you can copy data from serial monitor and paste directly on worksheet of excel.

We checked device by determining exothermic dissolution enthalpy for NaOH in water. For that, we ran the code and measured the temperature of 100 mL of water during 190 s. After that, we added 8.75 × 10^−2^ mol of NaOH_(s)_ and we continued measuring the temperature until complete 600 s. When sketch was finished, we copied data from “monitor series” window and pasted them in a worksheet of Excel and we proceeded to create the thermogram. The experimental protocol used in this work was similar to published previously in chemistry textbooks (Beran, 2014; Nelson et al., 2015; Zumdahl et al., 2017). Usually, the temperature measurements are to be made in changes as small as possible, mainly because the thermodynamic equations used in calorimetry are grounded on infinitesimal changes in temperature and reversibility so small changes in temperature are as close as possible to this reversibility framework.

## 3 Results

### 3.1 Testing device

**Figure 7:**
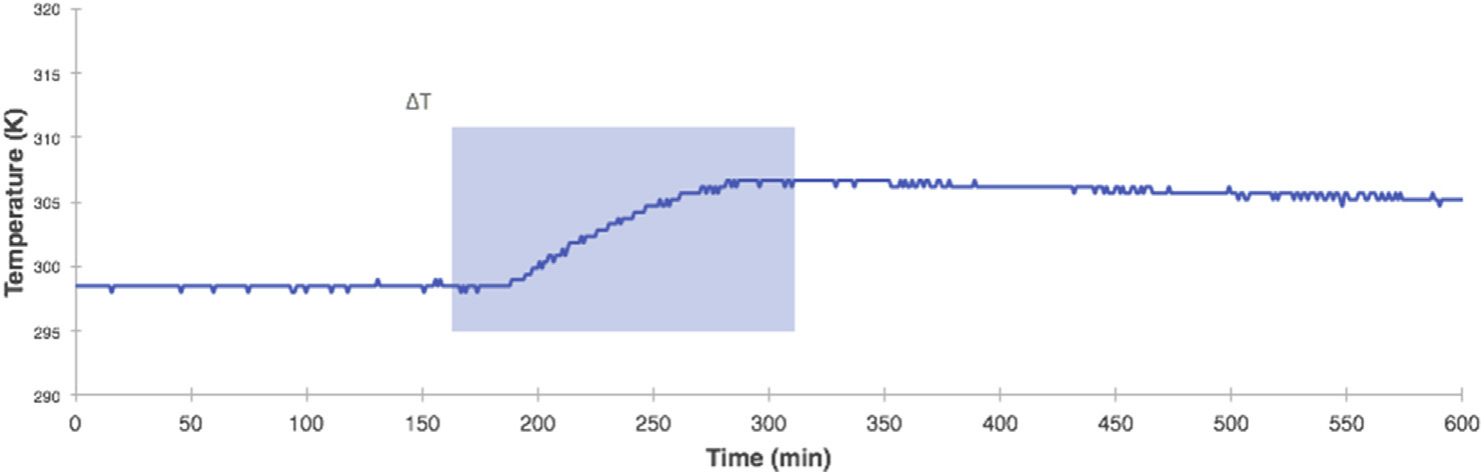
Data collected from temperature sensing device shown in Figure 4 the thermogram shows temperature vs. time; data acquisition took 600 s and we used one (1) second as delay.

The Figure 7 shows thermogram generated by the device after the dissolution of 7.50 × 10^−2^ (±0.001) moles of NaOH in 100 mL of water. Heat balance for dissolution process states:

(1) $$q_{dissolution}\;+\;q_{solution}\;=\;0\;$$

Where *q*_*dissolution*_ is heat transfer during NaOH dissolution process (exothermic process) and *q*_*solution*_ is heat absorbs by water (endothermic process); the quantity of heat gain by water is determined as follows (Brown et al., 2012; Zumdahl et al., 2017):

(2) $$q_{solution}\;=\;m_{water}\;\;x\;\;C_{water}\;\;\text{x }\;\text{ΔT}$$

Where *m*_*water*_ is mass water, *C*_*water*_ is the specific heat of water (4.18J∗K^−1^∗g^−1^) and ΔT is the increase in temperature, from Figure 7 we obtained ΔT = 8.8 (±0.5) K. If we assume that heat losses are negligible and because the process occurs at constant pressure you can determine Δ_*dis*_H for the process (Brown et al., 2012; Zumdahl et al., 2017):

(3) $${\text{Δ}}_{dis}H=-\frac{q_{solution}}{mol\;NaOH}$$

The solution of (3) for the test measurements gives -42.0 (±2.3) kJ∗mol^−1^. The experimental results had a 2.0 % error in comparison with the theoretical value, the percent error compares these results with the published value 43.0 kJ∗mol^−1^, (Zumdahl et al., 2017), demonstrating that this open-source device is a suitable option to be incorporated in teaching laboratories.

### 3.2 Discussion

The most effective method in gaining chemistry related knowledge is experimental and laboratory work, the basic science concepts are introduced by experiments, and besides the understanding of chemical concepts and processes can be increased if we provide new strategies (*e.g.* DIY-methodology) to practice new concepts in labs (Herga and Dinevski, 2012). Currently the modern equipment construction and design often rely on a “black box” construction philosophy, pushing the students and teachers away from the principles of operation of the machine, however, with the rise of the DIY culture, powerful prototyping platforms have become affordable and accessible for everyone (Meloni, 2016).

The device building in this work is practical and useful to introduce students to basics concepts of microelectronics and temperature sensing and besides, the DIY methodology gives to students the opportunity for enhancing the creative, aesthetic, and personal dimensions of students' scientific inquiries. The merits of the instrument-building tradition go beyond the immediate needs of research, it gives new tools to students to understand new basic concepts in different ways to traditional teaching (Resnick and Robbie Berg, 2000).

Kubínová et al., reported for the first time the term “ChemDuino” (a portmanteau of “chemistry” and “Arduino”), for general practice of applying the Arduino hardware and software (e.g., Wiring and OneWire) to improve chemistry teaching and learning (Kubínová and Šlégr, 2015). They described elsewhere the hardware required to build various instruments: thermometers with a range of −80 to 120 °C and −200 to 1400 °C; a pressure gauge; and a pH meter (Kubínova & Šleǵr, 2015). In our case to implemented the temperature sensor (LM35) wired to Arduino UNO to construct a first prototype of both cheap and practical calorimeter four using the DIY methodology. In market, you find a variety of configurable temperature sensors, Table 1 lists parameters for typical temperature sensors. The LM35 is a precision integrated-circuit temperature device with an output voltage linearly-proportional to the Centigrade temperature. The LM35 device has an advantage over linear temperature sensors calibrated in Kelvin, as the user is not required to subtract a large constant voltage from the output to obtain convenient Centigrade scaling. The low-output impedance, linear output, and precise inherent calibration of the LM35 device makes interfacing to readout or control circuitry especially easy (Texas Instruments, 1999). Comparing with other option in the market, the LM35 device is the cheap and easy for incorporating into the chemistry labs (see Table 1).

**Table 1:** List of temperature sensors using in typical devices for obtaining the temperature data during testing (Kubínová and Šlégr, 2015).

Temperature sensor	Features	Price (US $)	Brand/reference
Go Direct Temperature probe	-40 to 125 ± 0.25 °C, Connections: Bluetooth and USB	97	Vernier datasheet (Vernier, 2018)
Thermocouple	-50 to 700 ± 1 °C, range of 6.4–54.9 mV.	45	Datasheet (Tempsens, 2015)
DS18B20	-55 to 120 °C ±0.5 °C, with 10 mV/°C gain factor	2	Datasheet (Semiconductor Corp, n.d.)
TMP236	-55 to 120 °C ±1 °C, with 19.5 mV/°C gain factor	3	Texas instruments, datasheet (Texas Instruments, n.d.)
LM 35	-50 to 155 °C ±0.5 °C, with 10 mV/°C gain factor	1	Texas instruments, datasheet (Texas Instruments, 1999)

If we compare this device with traditional tools in the labs, this alternative offers some advantage: (i) conventional mercury thermometers are commonly used unto chemistry introductory labs of chemistry and chemical engineering, they are cheap but *in-situ* data acquisition of temperature is not possible, (ii) the commercial microcontrollers (e.i. National Instruments USB-data acquisition (DAQ)) gives better supporting for *in-situ* data acquisition but they are more expensive than Arduino microcontrollers and besides, the software of code programming is not for free, (iii) Texas Instruments offers many laboratory instrument options in different fields of chemistry, comparing to Arduino, for temperature sensors, the only disadvantage is the cost of the hardware (Texas Instruments, n.d.), (iv) Vernier durable sensors and high-quality classroom and laboratory options, the only disadvantage is the cost of the hardware and software, commonly the probes require the Vernier software to operate in optimal conditions (Vernier, 2018), (v) Phyton is another alternative as open-source software, it is a programming language (Python, n.d.), the raspberry pi is a microcontroller that supports Python code (Raspberry Pi, 2019), they gives same advantages than Arduino software and hardware. The device building in this work offers an alternative between different options available in the market and the main advantages are: (i) the device is suitable (it was stable and provides durability and precision in temperature data acquisition, the device was continuously tested for 8 months before we submitted the report for publication), (iii) cheap, the complete system had a total cost under $40, besides, the low cost of the device creates an alternative when financial aspect is relevant to develop applications to introduce into labs and classroom, (iii) it represents a very useful tool at the level of experimental practices for using DIY methodology for its construction.

The students can add further modifications for the calorimeter construction you have some option: (i) the total cost of device could be reduced replacing the aquarium pump air stirring by a mechanical stirring, (ii) the students can modify code of the sketch according to their requires, (iii) if the students have access to Office 365 (O365), they can use “data streamer” using Microsoft Excel software to visualize and analyze data, the data streamer provides students with a simple way to bring data from the physical world in and out of Excel's powerful digital canvas (Microsoft, 2020).

Finally, the device can be used to determine heat capacity and neutralization enthalpies, to verify Hess's law, and to demonstrate typical thermochemical experimental procedures in first-year undergraduate courses, including this device is especially useful for measuring cooling curves for phases diagrams. The device increases the teacher's options to introduce microelectronic basic concepts and temperature sensing in secondary courses and introductory chemical labs for Engineering and Chemistry degree.

## 4 Conclusions

We described the construction of an inexpensive and suitable calorimeter device for temperature sensing, reaction agitation system and data acquisition for typical thermochemical tests. In addition, we detailed circuit diagrams and all technical details to wiring LM35 to Arduino hardware, and calorimeter fabrication. Diagrams, photographs, and equipment program source codes, as well as instructions on uploading code to the Arduino software are available. During the building device, the students must be careful with the LM35 wiring to Arduino to ensure the correct operation of the device. The students can add further modifications for the calorimeter construction (e.g. sketch code, materials building, stream real-time data for O365 users). Comparing with traditional tools in the labs, the device offers some advantage as cost, versatility and reliability, the testing device is stable to be used in basic scholars and first-year undergraduates in introductory Chemistry and Chemical Engineering courses, the device was built using the DIY-method, and the complete system had a total cost under $40. Finally, the device is useful to introduce students to basics concepts of microelectronics and temperature sensing.

## Declarations

### Author contribution statement

William A Vallejo Lozada, Catalina Fajardo: Conceived and designed the experiments; Performed the experiments; Analyzed and interpreted the data; Contributed reagents, materials, analysis tools or data; Wrote the paper.

Carlos Diaz-Uribe: Conceived and designed the experiments; Analyzed and interpreted the data; Contributed reagents, materials, analysis tools or data; Wrote the paper.

### Funding statement

This work was supported by Universidad del Atlántico (Univesidad del Atlántico, RES. RECT. 003512 - 2019).

### Competing interest statement

The authors declare no conflict of interest.

### Additional information

No additional information is available for this paper.

## Appendix A Supplementary data

LM35 LM35
